# Taurine prevents mitochondrial dysfunction and protects mitochondria from reactive oxygen species and deuterium toxicity

**DOI:** 10.1007/s00726-024-03440-3

**Published:** 2025-01-10

**Authors:** Stephanie Seneff, Anthony M. Kyriakopoulos

**Affiliations:** 1 Computer Science and Artificial Intelligence Laboratory, Massachusetts Institute of Technology, Cambridge, MA 02139 USA; 2 Laboratory of Molecular Biology and Immunology, Department of Pharmacy, University of Patras, 26500 Rio-Patras, Greece

**Keywords:** Taurine, Mitochondrial dysfunction, Deuterium, Short chain fatty acids, Gut microbiome, Cancer

## Abstract

Taurine, although not a coding amino acid, is the most common free amino acid in the body. Taurine has multiple and complex functions in protecting mitochondria against oxidative-nitrosative stress. In this comprehensive review paper, we introduce a novel potential role for taurine in protecting from deuterium (heavy hydrogen) toxicity. This can be of crucial impact to either normal or cancer cells that have highly different mitochondrial redox status. Deuterium is an isotope of hydrogen with a neutron as well as a proton, making it about twice as heavy as hydrogen. We first explain the important role that the gut microbiome and the gut sulfomucin barrier play in deuterium management. We describe the synergistic effects of taurine in the gut to protect against the deleterious accumulation of deuterium in the mitochondria, which disrupts ATP synthesis by ATPase pumps. Moreover, taurine’s derivatives, N-chlorotaurine (NCT) and N-bromotaurine (NBrT), produced through spontaneous reaction of taurine with hypochlorite and hypobromite, have fascinating regulatory roles to protect from oxidative stress and beyond. We describe how taurine could potentially alleviate deuterium stress, primarily through metabolic collaboration among various gut microflora to produce deuterium depleted nutrients and deuterium depleted water, and in this way protect against leaky gut barrier, inflammatory bowel disease, and colon cancer.

## Introduction

Taurine is a conditionally essential amino acid in human metabolism that has many unique properties that set it apart from the other amino acids. It is the only amino acid that has a sulfonyl group replacing the carboxyl group. It is found in high concentrations in animal-based foods, such as meat, eggs, dairy products, shellfish and fish, but is not present at all in any plant-based foods except for Japanese seaweed (Laidlaw et al. 1990). It is conditionally essential because human cells can synthesize it from precursors such as methionine and cysteine, two other sulfur-containing amino acids. Taurine is not a coding amino acid, but it is the most common free amino acid in the body. It is stored in high concentrations in tissues with high metabolic demand, including the liver, brain, eyes, heart and skeletal muscles. Taurine acts as an osmolyte, regulating cell volume (Ripps and Shen 2012). It is actively transported into the mitochondria, where it buffers their high pH (Hansen et al. 2010).

Taurine protects the tissues from oxidative damage when neutrophils release high concentrations of the highly reactive molecule, hypochlorite (HOCl), in response to inflammation or an infection. HOCl reacts with taurine to produce N-chlorotaurine (NCT; also known as taurine chloramine), which has powerful signaling capabilities to help control inflammation (Olszanecki & Marcinkiewicz 2004). Taurine cannot be metabolized by human cells, but gut microbes are able to break it down to release sulfite, which then gets oxidized to sulfate anions that become available to support the synthesis of sulfomucins (Eichhorn et al. 1997). The liver delivers taurine to the gut microbes bound to bile acids, which facilitate microbial uptake and metabolism (Duszka 2022). Taurine deficiency is associated with many health issues, including cardiomyopathy, renal dysfunction, developmental abnormalities, and severe damage to retinal neurons (Ripps and Shen 2012).

Taurine protects against many diseases linked to mitochondrial defects, such as aging, metabolic syndrome, cancer, cardiovascular diseases and neurological disorders, although it is not easily oxidized and cannot act directly as a free radical scavenger (Jong et al. 2021). Taurine improves mitochondrial production of ATP, alleviates hypertension due to insulin resistance, and enhances exercise capability in rats (Rahman et al. 2011). Taurine’s powerful protective roles in mitochondria mostly relate to normalization of ATP production, which subsequently protects against atherosclerosis, in part through enhancement of cholesterol degradation by the liver by promoting bile acid secretion (Zaric et al. 2020). Taurine is released from cardiomyocytes during ischemia following acute cardiac injury, in proportion to the degree of insult (Kavianipour et al. 2004). Taurine depletion in cardiomyocytes results in aberrant increase of cytochrome C in the cytosol and apoptosis of cells, due to diminished respiratory chain function and reduced ATP production (Jong et al. 2011).

In this paper, we review the myriad ways in which taurine protects against human disease. A novelty of our work involves investigations around the role of deuterium (heavy hydrogen) in human disease. Clinicians are increasingly becoming aware of the potential benefits of deuterium depleted water (DDW) as a therapy for multiple diseases and conditions. A recent review paper showed that deuterium depletion is of therapeutic benefit in cancer prevention and treatment, depression, diabetes, and cognitive decline (Korchinsky et al. 2024). A study based on comparative data from the 50 states in the United States revealed a statistically significant inverse correlation between the amount of deuterium in the municipal water supply and depression rates (Strekalova et al. 2015). A study published in 2019 showed that DDW supplementation in rats not only caused a statistically significant decrease in the amount of deuterium in their blood, but also provided protection against acute hypoxia (Basov et al. 2019). When mice transplanted with a breast cancer cell line were fed foods that were substantially depleted in deuterium, cancer growth was slowed down (Somlyai et al. 2023).

Here, we present a novel view that the gut microbes play an essential role in providing deuterium depleted (deupleted) nutrients, especially, butyrate, to the host colonocytes forming the gut barrier. We propose that sulfomucins synthesized by goblet cells not only protect the barrier from pathogens, but also trap and sequester deuterium, thus reducing mitochondrial deuterium levels, resulting in improved mitochondrial health. We present a model for taurine utilization by the gut microbes to enhance the supply of both sulfate and heme to the colonocytes, essential nutrients for deuterium homeostasis and intestinal health.

## Deuterium toxicity and coping strategies in cellular physiology

In this section, we briefly review the role of deuterium, a natural heavy isotope of hydrogen, in mitochondrial dysfunction, and the coping strategies devised by biological organisms to prevent deuterium damage. Accumulation of deuterium in the mitochondria results in various toxic effects, especially through disruption of oxidative phosphorylation. Intestinal microbial flora synthesize deuterium depleted nutrients that maintain gut health and protect against colon cancer.

### Deuterium is toxic to mitochondria

Mitochondria are crucial organelles in human cells, and they produce the majority of the ATP used by the cell as its energy currency. Mitochondrial dysfunction is associated with many chronic diseases, including inflammatory bowel disease (IBD) and colon cancer (Kos and Dabravolski 2021). Impaired mitochondrial oxidative phosphorylation results in the release of large amounts of reactive oxygen species (ROS), along with a loss in the efficiency of ATP production.

F0F1 ATPase (F-ATPase) is a molecular motor positioned in the inner membrane of the mitochondrial intermembrane space that uses proton motive force as protons cross the membrane to generate the energy needed to phosphorylate ATP, while producing water from oxygen molecules. Protons moving through the ATPase pumps need to dissociate rapidly from Asp61 in F0 as an essential part of the catalytic reaction. Deuterons bind more tightly to organic molecules, and dissociate more slowly, resulting in a stutter in the pump (Olgun 2007; Boros et al. 2016).

The γ c-terminal helix of the ATPase molecular motor is the tip of the rotor that drives ATP synthesis. A hydrogen–deuterium exchange experiment showed that this helix accumulates significantly more deuterium from heavy water than other parts of the enzyme complex. Rotation of the γ rotor caused greatly enhanced deuteration in this c-terminal helix. The rotor tip is much less stable than other helices in the protein, and excess deuterium causes it to unfold. Deuterium weakens hydrogen bonds, and their breakage likely induces a stutter in the motor rotation (Murcia Rios et al. 2018). Repeated stutters can be highly disruptive of the ATPase function, leading to inefficiencies in ATP production and increases in ROS (Hansen et al. 2006; Ripps and Shen 2012). These ROS can lead to mitochondrial DNA damage, which is carcinogenic if the damage is not adequately repaired. As a result of the high sensitivity of ATPase pumps to deuterium, metabolic policies have developed sophisticated strategies to minimize the number of deuterons that are pumped into the intermembrane space, relative to protons (Seneff 2024).

When cells are exposed to heavy water, their ability to repair double-strand breaks in DNA is significantly impaired. Many enzymes involved in DNA repair use proton tunneling to facilitate the reaction, and, as a consequence, they exhibit a high deuterium KIE. Deuterons, being twice as heavy, have reduced quantum-level mechanisms required for efficient quantum tunneling. This significantly slows down the reaction rate of these enzymes, leaving DNA damage unrepaired (Yasuda et al. 2024).

### Gut microbes, butyrate, and deupleted hydrogen gas

Butyrate is a short chain fatty acid produced by anaerobic bacteria in the gut. Many studies have shown that butyrate is protective against IBD and colon cancer (Arise et al. 2022; Kamierczak-Siedlecka et al. 2022). Treatment of mouse colonic epithelial cells with butyrate stimulated expression of N-acetylglucosamine 6-O-sulfotransferase-2 (GlcNAc6ST-2), an essential enzyme for the synthesis of sulfomucins (Tobisawa et al. 2010). Hydrogen gas is used by certain gut microbes as a reducing agent to convert carbon dioxide into acetate, a precursor to butyrate. High concentrations of hydrogen gas in the gut promote butyrate synthesis (Campbell et al. 2023). The source of the hydrogen gas can be traced to the action of microbial hydrogenases during fermentation.

A seminal paper published in 1960 measured the amount of deuterium present in hydrogen gas produced by a strain of *Pseudomonas* marine bacterium. This microbe synthesized hydrogen gas (H_2_) that was 80% depleted in deuterium, compared to the normal amounts present in water, an astounding achievement. The gas was produced, along with carbon dioxide (CO_2_), from formate. The enzymes that produced the hydrogen gas are similar to hydrogenase enzymes present in *Esherichia coli* and other coliform bacteria that populate the human gut (Krichevsky et al. 1961). This phenomenon can be explained by the fact that many hydrogenases exhibit a very large deuterium KIE, as high as 43 in an acidic environment, which has been shown experimentally for the microbial class of [NiFe]-hydrogenases (Greene et al. 2015). This means that the extraction of a proton from the active site would be 43 times more efficient than extraction of a deuteron. According to Greene et al., (2015) “The large KIE at acidic pH can only be reconciled by an EPT [electron-proton transfer] rate-determining step involving proton tunneling.” *p.* 4562.

### Exclusion zone water and deuterium

According to studies by Prof. Gerald Pollack and his team, highly sulfonated polymers have remarkable effects on the structure of the surrounding water, creating gelled water that excludes solutes, known as “Exclusion Zone” (EZ) water (Zheng & Pollack 2003; Zheng et al. 2006). His research team has conducted several experiments involving Nafion, a sulfonated tetrafluoroethylene-based fluoropolymer that can simulate the effects of sulfated proteoglycans which are prevalent in human tissues (Hwang et al. 2018). This model could reasonably be applied to the sulfomucins lining the gut barrier (Cornick et al. 2015). Pollack and others have maintained that EZ water mobilizes protons and becomes negatively charged, releasing protons into the surrounding fluid water (Seneff & Nigh 2019). Although Pollack’s work is somewhat controversial, a recent review paper acknowledges that the concept is valid, while the exact mechanisms are yet to be worked out in detail (Elton et al. 2020).

Briefly, deuterium adsorbs competitively on the sulfonic groups in Nafion compared to hydrogen. The polymer fibers unwind into the bulk of the adjoining water upon swelling. These unwound fibers form a vastly extended brush-type border, projecting out from the membrane surface, reminiscent of the brush-like fibers that form in the endothelial glycocalyx (Kabedev & Lobaskin 2018). A remarkable experiment demonstrated that, if the water next to a hydrophilic Nafion surface is replaced with deupleted water (3 ppm), the fibrils no longer form (Ninham et al. 2023).

The inflammation associated with vascular disease promotes the oxidation of serum homocysteine to yield sulfate, which can then reinforce the sulfate population in the glycocalyx, strengthening the EZ water zone and improving blood flow (Seneff et al. 2015). This implies that sulfate deficiency in the glycocalyx is a causal factor in vascular disease (Seneff & Nigh 2019). Similarly, we suggest here that a deficiency in the sulfomucins lining the gut barrier compromises barrier function. We further explore the relevance of this phenomenon to taurine’s mitochondrial protection.

Biological organisms can fractionate deuterium so as to create deuterium disequilibrium in different compartments, such as the extracellular space and the mitochondria (Boros et al. 2024). It is tempting to speculate that sulfated proteoglycans (containing heparan sulfate, chondroitin sulfate, dermatan sulfate, keratan sulfate, etc.) that make up the extracellular matrix of most human cells play a significant role in trapping deuterium within the gelled water matrix, while releasing deupleted protons into the surrounding fluid water – the lumen in the gut or the circulating blood in the case of endothelial cells. This hypothesized phenomenon may be an important way in which the organism reduces the amount of deuterium reaching the mitochondria, through its sequestration outside the cell (Seneff 2024).

### Dehydrogenases supply deupleted protons to the ATPase pumps

There are many enzymes localized to the mitochondrial matrix that facilitate the delivery of deupleted protons to power the ATPase pumps. Ultimately, the main supplier of protons is reduced nicotinamide adenine dinucleotide (NADH). The enzyme NADH dehydrogenase (Complex I) pumps protons from NADH into the intermembrane space to build up the proton gradient. A large number of other dehydrogenases are localized to the mitochondria, and many of them convert NAD + to NADH by extracting protons from a source molecule.

Most dehydrogenases are members of a class of enzymes called flavoproteins, which are known for their ability to exploit proton tunneling to get around an energy barrier. Deuterons are much less competent at tunneling due to their larger mass. As a result, flavoproteins tend to have a high deuterium KIE, ranging from 3.5 to 10, and it can be as high as 25 (Hay et al. 2009). Other dehydrogenases in the mitochondria besides NADH dehydrogenase include α-ketoglutarate dehydrogenase, isocitrate dehydrogenase, succinate dehydrogenase, and malate dehydrogenase in the TCA cycle, as well as several acyl-CoA dehydrogenases that catalyze the first step of mitochondrial fatty acid *β*-oxidation.

Glutathione likely plays an important role, not only in quenching reactive oxygen, but also in supplying deupleted water to the mitochondrial matrix. The phosphorylated form of NADH, NADPH, is needed to restore reduced glutathione (via glutathione reductase) following glutathione oxidation to form glutathione disulfide (GSSG), quenching ROS. The two water molecules that are produced from hydrogen peroxide (H_2_O_2_), in the reaction catalyzed by glutathione peroxide, can thus be traced back to NADPH, and can be expected to be deupleted, because NADPH in the mitochondria is derived from NADH via the mitochondrial enzyme NADH kinase (Zhu et al. 2021).

## Taurine’s main roles in human physiology

Taurine protects mitochondria against oxidative stress in multiple ways. Moreover, the role of taurine in mitochondria involves conjugation with tRNAs and in this way protection from mitochondrial respiratory failure that can result in severe disease. A role for taurine to protect against cancer can be explained by alleviation of deuterium toxicity through taurine’s influence on gut microbial metabolism.

### Taurine’s buffering effects in mitochondria

The mitochondrial matrix needs to maintain a relatively high and stable (alkaline) pH (typically around 7.8) in order to sustain the proton gradient that drives ATPase synthesis of ATP via proton motive force as protons exit the intermembrane space via the ATPase pumps. Furthermore, critical enzymes in the matrix work optimally at this alkaline pH. Under optimum conditions of mitochondrial functioning, there should be a stabilized pH gradient and membrane potential across the inner mitochondrial membrane to maintain a slightly alkaline environment in the mitochondrial matrix. This helps maintain important enzyme activities involved in the reactions of the tricarboxylic acid (TCA) cycle (Arnold & Finley 2023). Among these enzymes are the Acyl-CoA Dehydrogenases (ACADs) which catalyze the *α,β*-oxidation step of fatty acids (dehydrogenation of acyl-CoA esters), and are also tightly involved in amino acid catabolism (Swigonová et al. 2009).

Most of the taurine inside a cell is localized to the mitochondrial matrix, where it buffers its high pH (Hansen et al. 2006). Metabolizing acetyl-CoA derived from lipid *β*-oxidation produces CO_2_, which increases acidity, and taurine buffer protects from this effect (Hansen et al. 2006). The brain and heart store large amounts of taurine, due to their strong reliance on oxidative phosphorylation in the mitochondria. With pKα values at 9.0 (25°C) and 8.6 (37°C), taurine helps to maintain the alkaline environment (buffering capacity to maintain a pH between 7.5 and 8.5) in the matrix, so that important mitochondrial enzymes function efficiently (Hansen et al. 2010). Of particular importance is Acyl-CoA Dehydrogenase Short Chain (ACADS), the enzyme that catalyzes the initial step in mitochondrial *β*-oxidation of short chain fatty acids. Taurine’s buffering activity in the mitochondrial matrix is very important to maintain homeostasis during oxidative stress, as for instance in immunometabolism, and thus, it limits inflammation (Ryan & O'Neill 2020).

However, taurine’s buffering capacity goes beyond the stabilization of enzymatic activities during oxidative stress. While taurine acts as a pH buffering molecule, it also facilitates protection by the strong antioxidant scavenger glutathione (GSH). The optimum pH for enzymatic activity of glutathione peroxidase is 8.5 (Awasthi et al. 1975), and so taurine supports its activity by maintaining this high pH in the mitochondrial matrix. Taurine, by buffering an alkaline pH during increased oxidation, helps maintain the redox equilibrium of the NADH/NAD^+ ^redox pair conferred by the GSH/GSSG pair. In this way, it is not only the alkaline pH which is maintained in the mitochondrial matrix, but, importantly, due to taurine, the redox buffer GSH further stabilizes the membrane potential during alarming oxidative conditions (Hansen and Grunnet 2013).

Cardiomyocytes subjected to severe taurine deficiency suffered from impaired respiratory chain function and oxidative stress, which activated the mitochondrial permeability transition (MPT) pore and induced apoptosis (Jong et al. 2011). The opening of this pore rapidly acidifies the matrix, inducing the release of cytochrome C and apoptosis.

GSH not only reduces radical oxygen species (ROS) during oxidative stress, but it also assists in the production of deupleted water in the mitochondria. The spontaneous oxidation of two GSH molecules to produce GSSG in the presence of hydrogen peroxide yields two molecules of water (Townsend et al. 2003), and these are deupleted because the -SH moiety in glutathione derived its hydrogen atom from mitochondrial NADPH. Experimental evidence has shown that a sulfhydryl group (-SH) attached to cysteine does not easily exchange its proton with a deuteron from the water (Rozman 2005).

Furthermore, taurine, acting as a potent osmolyte, also helps to maintain the intracellular pH during a regulatory cell volume decrease. Under conditions of osmotic stress, there is a massive (50%) co-transportation of taurine as well as Na^+^ outside the cell which evidently leads to the maintenance of the intracellular content of Cl^–^ ions and pH. This change of cellular taurine permeability helps to maintain a constant intracellular pH and membrane potential, and also protects the plasma membrane from damage due to excessive stretching (Guizouarn et al. 2000).

### Taurine conjugation to mitochondrial tRNAs

Taurine plays a fascinating and still poorly understood role in the mitochondria by conjugating to certain transfer RNAs (tRNAs). Mitochondrial Encephalopathy Lactic Acidosis and Stroke-like episodes (MELAS) syndrome emerges due to taurine-related defects in mitochondria (Jong et al. 2021). Taurine conjugates with the mitochondrial tRNALeu(UUR) to produce taurino-methyl-uridine derivatives. Uridines not modified by taurine have a weaker affinity for the UUG anticodon, and this results in inefficient synthesis of important mitochondrial proteins. Specific mutations in the tRNALeu(UUR) gene alter the normal taurine-containing conjugated modification at the anticodon positions (wobble positions). These mutations are found only in MELAS patients and not in other patients that have mitochondrial disease but do not show MELAS-related symptoms (Kirino et al. 2005).

Apart from the genetic mutations, other experiments have proven that it is the lack of taurine conjugation to the tRNALeu(UUR) that leads to MELAS syndrome. The symptoms of taurine deficiency overlap significantly with MELAS syndrome, where both conditions lead to metabolic abnormalities, growth failure, exercise intolerance, and renal defects, reflecting impaired function of mitochondrial complex I (Schaffer et al. 2013). Low taurine conjugation to the tRNALeu(UUR) simply due to taurine content depletion in mitochondria results in the reduction of proteins that are essential in proper mitochondrial function – NADH-ubiquinone oxidoreductase chains 5 and 6 (ND5 and ND6), critical components of complex I (Jong et al. 2010). Lactic acidosis (LA), a feature of MELAS syndrome, arises due to impaired tissue oxygenation, causing overproduction of lactate (Andersen et al. 2013).

*β*-alanine is a non-essential naturally occurring amino acid that is used as a supplement to boost athletic performance (Bellinger 2014). However, there is some concern that it may disrupt taurine uptake into cells, because it is taken up via the taurine transporter. An experiment conducted by CJ Jong et al. (2010) investigated whether and how β-alanine might impact ND6 synthesis rates. Curiously, *β*-alanine treatments did not result in a significant reduction of taurine levels in the mitochondria, yet it still led to reduced synthesis of ND6. Although this appears to be a paradox, current research data show that, apparently, taurine and β-alanine act as antagonists and *β*-alanine presumably interferes directly with taurine conjugation to tRNA (Shetewy et al. 2016).

ND6 is needed for the proper transport of electrons across the respiratory chain. Reduced ND6 expression leads to less oxygen and water produced through the respiratory complex I – complex III chain reactions (Loschen et al. 1971; Brand 2016). Hence, free radical oxygen species (e.g., O_2_·^–^) will remain available to react with nitrogenous compounds to form peroxynitrite (ONOO^−^), which is highly reactive (Sies et al. 2017).

Summarizing, the buffering effect of taurine inside the mitochondrial matrix is equally important for the preservation of the inner mitochondrial membrane potential and for safeguarding important matrix enzyme functions. Taurine’s protection against mitochondrial disease associated with MELAS syndrome involves its conjugation to the uridines of tRNALeu(UUR), needed to provide the right anticodon matching, and, if this conjugation ability is lost, important mitochondrial protein expression like that of ND5 and ND6 is also deregulated. The loss of these mitochondrial protein activities leads to acceleration of oxidative and nitrosative stress.

### The complex roles of taurine in cancer cells

Cancer cells upregulate enzymes that support increased synthesis of NADPH at the expense of NADH. Mitochondrial NAD kinase, overexpressed in many cancers, converts NAD^+^ to NADP^+^, consuming ATP (Rather et al. 2021). Cancer cells depend upon *β*-oxidation of both endogenous and exogenous fatty acids to maintain their supply of NADPH. Isocitrate, derived from fatty acid *β*-oxidation, is converted to oxoglutarate (α-ketoglutarate) by mitochondrial isocitrate dehydrogenase, which generates NADPH from NADP^+^ (Reitman & Yan 2010). Mitochondrial NADPH, in turn, is essential for restoring 2GSH from GSSG, i.e., for maintaining glutathione in its reduced state (Pike et al. 2011). Furthermore, fatty acids are deupleted nutrients, so sourcing NADPH from fatty acids helps assure an increased ratio of ^1^H over ^2^H in the mitochondrial water produced when glutathione reacts with H_2_O_2_ (Korchinsky et al. 2024). This may be the primary reason why cancer cells overexpress the taurine transporter SLC6A6 (Stary & Bajda 2023), because taurine can assure the high pH in the matrix that promotes *β*-oxidation of fatty acids. Human glioblastoma cells responded to inhibition of fatty acid oxidation with increased ROS, ATP depletion, and cell death (Pike et al. 2011). Therapeutic knockdown of the taurine transporter renders cancer cells more susceptible to chemotherapy-induced apoptotis (Han 2019).

Another possible benefit to cancer cells of aggressive taurine uptake is that the taurine supply to the resident immune cells becomes depleted. A study on programmed death 1 (PD-1) blockade in lung cancer patients revealed that those with higher serum taurine levels responded better to treatment. The authors hypothesized that abundant taurine improved the ability of tumor resident CD8^+^ T cells to clear the cancer. They confirmed this hypothesis by culturing CD8^+^ T cells in taurine-supplemented medium. This resulted in enhanced T-cell proliferation and increased secretion of cytotoxic cytokines (Ping et al. 2023).

Taurine reacts spontaneously with the highly reactive molecules, HOCl and HOBr to respectively produce N-chlorotaurine (NCT) and N-bromotaurine (NBrT). HOCl and HOBr are released by eosinophils and neutrophils under inflammatory conditions via the enzymes eosinophil peroxidase and myeloperoxidase (MPO) respectively (Thomas et al. 1995; Henderson et al. 2001). NCT and NBrT are far less reactive, and they are also signaling molecules that can induce heme oxygenase 1 (HO-1) expression and subsequent phagocytosis in macrophages (Kim et al. 2010, 2018).

NCT produced from taurine during inflammation accumulates in mitochondria. Cells take up NCT via the taurine transporter SLC6A6, which, as we’ve seen, is often upregulated in cancer cells (Cao et al. 2024). NCT induces opening of the permeability pore and swells mitochondria in lymphoma cells, a condition that leads to cancer cell death by apoptosis (Klamt and Shacter 2005). By contrast, NCT is remarkably well tolerated by normal cells (Pilz et al. 2024), possibly due to reduced uptake compared to cancer cells (Cao et al. 2024).

It has been suggested that taurine’s oxidative derivatives, by activating HO-1, reduce oxidative stress during inflammation through the synthesis of bilirubin, a powerful antioxidant that may even be superior to glutathione (Marcinkiewicz et al. 2009; Kyriakopoulos et al. 2022). Cancer cells maintain a ten-fold higher level of NADP^+^ relative to NADPH in their mitochondria, compared to normal cells (Moreira et al. 2016). NCT, by upregulating HO-1, further increases the NADP^+^/NADPH ratio; however, a higher NADP^+^/ NADPH ratio may mean a lower synthesis of fatty acids that are needed not only for *β*-oxidation but also for the membranes of cancer cell clones. This can be an attributing factor to cancer cell death (Moreira et al. 2016). The de novo synthesis of fatty acids (FA) from cytoplasmic acetyl-CoA constitutes an important factor for cancer cell survival and growth (Snaebjornsson et al. 2020). Each time HO-1 reduces hemoglobin to biliverdin, one molecule of NADPH is converted to NADP^+^. Furthermore, each time biliverdin is reduced to bilirubin, another molecule of NADPH is lost. This happens repeatedly if significant amounts of H_2_O_2_ are being produced by deuterium-loaded mitochondria. Restoration of reduced glutathione also costs one molecule of NADPH. It is possible that, while bilirubin serves an important protective role to preserve mitochondrial health, a depleted supply of NADPH may prevent cancer cells from being able to maintain an adequate supply of lipids from de novo synthesis to meet their high demand.

### Taurine-conjugated bile acids and their interaction with gut microbes: Relationship to inflammatory bowel disease (IBD), ulcerative colitis (UC), and colon cancer

Bile acids are synthesized from cholesterol in hepatocytes and are then released to the duodenum as bile salts (BSs), following conjugation with either taurine or glycine, a process called bile acid amidation. The primary bile acids are called cholic acid and chenodeoxycholic acid. Taurine conjugation to bile acids depends on the amount of dietary taurine. Variations between glycine to taurine ratios of BSs are seen in different geographical populations that depend on their diet. In general, an animal fat-based diet favors taurine conjugation to BAs, whereas a vegetable-based diet favors glycine conjugation (Ridlon et al. 2016a). There is a specific association between differing ratios of taurine and glycine conjugations to BAs and disease (Nagana Gowda et al. 2009).

The glycine and taurine conjugated primary BSs are stored in the gallbladder and released into the intestines, to assist in the digestion of dietary fats by solubilizing them. After significant processing by the gut microbes, they are reabsorbed in the small intestine (terminal ileum) and return to the liver through the enterohepatic circulation. Only 5% of the BSs escape through the feces. Primary BSs are transformed by the gut bacteria into secondary BSs, such as deoxycholic acid. Conjugated with glycine, these become glycodeoxycholic acid (GDCA) and glcyohenodeoxycholic acid (TCDCA). Taurine-conjugated BSs become taurodeoxycholic acid (TDCA) and taurochenodeoxycholic acid (TCDCA). Another important secondary BS is lithocholic acid (Sheng et al. 2022). Current studies directly associate the metabolism of secondary BS products of intestinal bacterial colonizers with various diseases, ranging from hepatocellular and cholangiocellular carcinoma, to inflammatory bowel syndrome, to ulcerative colitis and colon cancer (Ridlon et al. 2016a; Fuchs & Trauner 2022).

Bacterial species of *Listeria*, *Clostridium*, *Bifidobacterium*, *Bacteriodes* and other genera are capable of deconjugating BSs using hydrolases, thus releasing free taurine, glycine and their respective BAs into the enteric environment (Ridlon et al. 2016b). Deregulation in these enteric bacterial transformations of BSs, for example, increased deconjugations of taurine BSs, can cause irregularities in sulfur metabolism in the colon and can lead to disease. The main reason is the endpoint over-production of hydrogen sulfide (H_2_S), which is a major causative factor in IBD and colorectal cancer (Carbonero et al. 2012).

The microbial intestinal colonizer, sulfidogenic *Bilophila wadsworthia* uses taurine for respiration. Taurine’s abundance due to, e.g., its increased deconjugation from primary and secondary BSs, can lead to *Bilophila wadsworthia* overgrowth and therefore to increased production of H_2_S, which induces inflammation and becomes toxic in excessive amounts, and can increase colon cancer risk (Ridlon et al. 2016a). Moreover, the transformation to deoxycholate of the cholic acid liberated by taurocholate deconjugation, by 7α-dehydroxylating *Clostridium* spp. is a strong primary pro-inflammatory signal that promotes the development of colorectal cancer (Ou et al. 2013).

In the study of J Ou et al. (2013), it was determined that production of the short chain fatty acid (SCFA) butyrate by butyrate-producing bacteria in the colon is the main factor that protects from mutagenic transformations and deconjugations of secondary bile acids that finally produce colon cancer. This depends on butyrate production by microbial fermenters in the large intestine and the amount of H_2_S being produced, as a result of taurine catabolism (Devkota et al. 2012). Other studies have shown an inverse relationship between butyrate content in the intestine and the development of UC and IBD (Recharla et al. 2023).

Butyrate is one of the primary sources of energy for colonocytes, together with glutamine and glucose. However, for this SCFA to become available for energy provision, it needs to become *β*-oxidized. Failure of butyrate oxidation is suggested to be an energy deficiency state that leads to UC (Roediger 1980). Moreover, inhibition of butyrate *β*-oxidation results immediately in acute colitis, showing that this is a direct inflammatory signal for the colonic mucosa (Roediger & Nance 1986). Finally, hydrogen sulfide impairs the function of SCFA – acetyl CoA dehydrogenases and prevents butyrate oxidation, compromising the intactness of colonic mucosa (Roediger & Nance 1986; Babidge et al. 1998).

It is, therefore, a matter of homeostatic balance between taurine’s important function in the digestion of food, the maintenance of normal deconjugation and transformation of secondary BAs by large intestine colonizers, and the normal production of nutritional SCFA metabolites like butyrate that determine the physiological state of the colonic mucosa. Colonic mucins overlay the mucosal epithelium and provide an important interface between colonic cells and the luminal contents (including the microbial flora). Mucins have a peptide backbone with alternating glycosylated and non-glycosylated domains. According to their terminal sialic acid or sulfate groups, they are categorized as sialomucins or sulfomucins respectively (Filipe 1979).

Regarding the production of H_2_S, sulfate reducing bacterial (SRB) genera, namely *Desulfovibrio*, *Desulfobacter*, *Desulfobulbus and Desulfotomaculum,* catalyse its production from the sulfomucin colonic layer (Croix et al. 2011). However, the production of H_2_S from anaerobic respiration of taurine by *Bilophila wadsworthia,* differs from that produced by SRB, as the sulfite reductase used in the taurine pathway is different in structure from that used by the SRB species (Carbonero et al. 2012). Both taurine reducing bacteria and SRB are regular suppliers of H_2_S by reducing the available sulfate and thus influencing the sulfur cycle in the human intestine. Microbial sulfur metabolism in the human gut may be more complicated than once thought. A plethora of microbial sulfidogenic genes associate with colon cancer. and ever more taurine sulfate reductive genes expressed by novel species are still being discovered in the human colon microbiome (Wolf et al. 2022).

The presence of increased sulfomucin proportion in the colon does not necessarily increase the colonic growth of SRB but instead alters its composition. Under increased sulfomucin proportion, the *Desulfibrio *spp., which are the main H_2_S producers, become reduced in population compared to the rest of the SRB genera, but this may have to do with its absolute metabolic requirements for respiration which can be other than sulfate alone (Croix et al. 2011). Nevertheless, there is clinical evidence implicating SRB, and especially *Desulfibrio,* with increased H_2_S production and association with UC (Gibson et al. 1991). This indicates again that the homeostatic equilibrium between the intestinal colonizers sets the requirements for a healthy or a diseased condition in the colon. This depends in part on the enzymes they produce to degrade sulfated compounds in the gut (Robertson & Wright 1997).

### Obligate anaerobes and sulfomucins

Sulfomucins are produced primarily by goblet cells, which are specialized columnar epithelial cells that are concentrated in areas of the colon that are densely populated with bacteria. These sulfomucins form a thick barrier that hosts microbes and keeps them apart from the epithelial surface, minimizing pathogenic infection. Germ free mice have a much thinner mucus barrier, testimony that there is a symbiosis between the microbes and the goblet cells. Some of the microbes can detach and consume the sulfomucins, while giving back deupleted SCFAs to the host cells in return (Deplancke & Gaskins 2001).

Obligate anaerobes play an important role in maintaining gut health, although they are difficult to study in vitro due to their sensitivity to oxygen (Maier et al. 2014). Two obligate anaerobes, *Faecalibacterium prausnitzii* and *Akkermansia mucinophila* are among the most abundant gut bacterial species (Lopez-Siles et al. 2017; Zhou 2017; Lee et al. 2022). These two species are often depleted in association with several gut disorders. Their extreme sensitivity to oxygen likely contributes to their reduction in numbers during conditions of oxidative stress.

*Akkermansia mucinophila* is a gram-negative anaerobic mucus-layer-degrading bacterium found in the human gut, normally representing 3–5% of the microbiota. As its name suggests, its primary source of nutrition is the sulfomucins produced by goblet cells in the colon. Its prevalence is inversely correlated with body weight, inflammation, metabolic syndrome, and both type 1 and type 2 diabetes. Leptin-deficient obese (ob/ob) mice had a 3500-fold reduction in *A. muciniphila* compared to their littermates. *A. municiphila* as a probiotic bacterium slows the progression of diabetes, obesity, and IBD in mice, and clinical studies on humans are showing promising preliminary results (Rodrigues et al. 2022).

Although it degrades sulfomucins, the abundance of *A. muciniphila* in the gut is strongly *positively* correlated with the amount of sulfated mucin, and there is also a strong inverse association between inflammatory markers and *A. muciniphila*. Inflammation leads to oxidative stress which would expose *A. muciniphila*, a strict anaerobe, to oxygen, to which it is highly sensitive (Earley et al. 2019). *A. muciniphila* likely increases the mucus turnover rate by producing deupleted SCFAs, primarily acetate and propionate, via hydrogen recycling through fermentation. In support of this theory, *A. muciniphila* supplementation increased the number of mucin-producing goblet cells in mice (Earley et al. 2019). Reduced amounts of sulfomucin compromises the gut barrier, leading to increased inflammation and also reduces the ability to trap deuterium in gelled water, increasing the deuterium levels in the gut lumen. An increased relative abundance of *A. muciniphila* in the gut was associated with a superior outcome in advanced non-small cell lung cancer patients treated with PD-1 blockade (Derosa et al. 2022).

*F. prausnitzii* is noteworthy for its ability to synthesize butyrate. It feeds on dietary fiber, which is otherwise undigestible. Butyrate is the dominant energy source for colonocytes, via *β*-oxidation and the TCA cycle. Like *A. mucinophila*, *F. prausnitzii* shows promise as a probiotic to treat multiple gut conditions (He et al. 2021). Since butyrate is synthesized from acetate, which in turn is derived from CO_2_ and H_2_ gases via the acetogenic bacteria, butyrate is a low-deuterium nutrient. High levels of H_2_ gas in the gut stimulates butyrate production (Campbell et al. 2023).

### Taurine oxidation in the gut: potential widespread benefit

Various biological pathways, with taurine playing a central role, involve a collaboration between gut microbes and the host cells. These pathways can support a process of healing from inflammation in the gut, while at the same time alleviating deuterium overload in the mitochondria.

It is conceivable that taurine-conjugated bile salts serve a purpose of positioning taurine near the membrane of microbes able to metabolize taurine. Many microbial species release bile salt dehydrolase into the medium, which could detach taurine from a bile salt embedded in the *E. coli* membrane (Larabi et al. 2023). *E. coli* strains have defense mechanisms against bile acids, including various efflux systems (Thanassi et al. 1997). They also express a protein called sn-glycerol-3-phosphate-binding periplasmic protein (UgpB) which has been found to moonlight as a protector against bile-induced protein aggregation in the periplasm (Lee et al. 2020). Thus, *E. coli* strains are well equipped to defend against bile acid toxicity.

The enzymes that metabolize taurine in bacteria are usually soluble, intracellular enzymes, so transport across the cell membrane is an essential first step in its metabolism (Cook and Denger 2006). *E. coli* possess a taurine cluster that contains four genes, referred to collectively as tauABCD. TauABC is concerned with transporting taurine into the cell, and tauD (taurine α-ketoglutarate dioxygenase) is a remarkable enzyme that can utilize dioxygen to oxidize taurine, yielding sulfite and aminoacetaldehyde, while simultaneously oxidizing α-ketoglutarate to succinate. TauD has a very high deuterium KIE (37-fold decrease with deuterium) for extraction of the C1 hydrogen from taurine (Price et al. 2003). *E. coli* strains do not express the genes that reduce sulfite to H_2_S, but they do possess a sulfite oxidase gene that depends on heme as a cofactor. Thus, they can also oxidize sulfite to sulfate, which is needed by goblet cells for sulfomucin production. TauD is not just restricted to *E. coli*, but is also expressed by many other bacteria, including *Enterobacter*, *Citrobacter*, *Morganella*, *Hafnia*, and *Raoultella* species, regular commensals and opportunistic pathogens of the human gut (Cook and Denger 2006).

Once sulfite is removed from taurine, it leaves behind aminoacetaldehyde, which can likely be further metabolized to glycine by several microbial originating variants of acetaldehyde dehydrogenase. While aminoacetaldehyde dehydrogenase appears to be rare in the microbial kingdom, four dehydrogenases synthesized by *Pseudomonas aeruginosa* have been found to be specific for aminoacetaldehyde, yielding glycine (Muoz-Clares et al. 2023). Since acetaldehyde dehydrogenases often show a broad substrate specificity, it is likely that other microbial acetaldehyde dehydrogenases could produce glycine by dehydrogenating aminoacetaldehyde.

Glycine and succinate are the two precursors needed to produce heme. Heme has many uses, as it is a cofactor in several important genes in both human cells and microbes, including, as we have described, sulfite oxidase. Heme is also a substrate for HO-1, which is stimulated by taurine through its NCT derivative, as already described (Gozzelino et al. 2010). HO-1 metabolizes heme to produce biliverdin, and, with the help of biliverdin reductase, bilirubin (Wegiel and Otterbein 2012). Bilirubin is an incredibly potent antioxidant, even superior to glutathione. Barañano et al. (2002) wrote: “The potent physiologic antioxidant actions of bilirubin reflect an amplification cycle whereby bilirubin, acting as an antioxidant, is itself oxidized to biliverdin and then recycled by biliverdin reductase back to bilirubin. This redox cycle may constitute the principal physiologic function of bilirubin.” Bilirubin reacts spontaneously with H_2_O_2_ to produce two molecules of DDW. The water produced through this reaction will be deupleted because its protons are sourced from NADH.

Neutrophils are the first immune cells recruited to fight a pathogenic infection, especially in the gut. They release MPO, a hemedependent enzyme, which produces HOCl, a powerful bactericidal agent, from H_2_O_2_. Taurine plays an important protective role through its spontaneous reaction with HOCl to form NCT (Arnhold 2020). NCT not only clears HOCl, but also induces increased expression of HO-1, as already described, and other antioxidant enzymes, through activation of the Nrf2 pathway (Sun Jang et al. 2009).

**Fig. 1:**
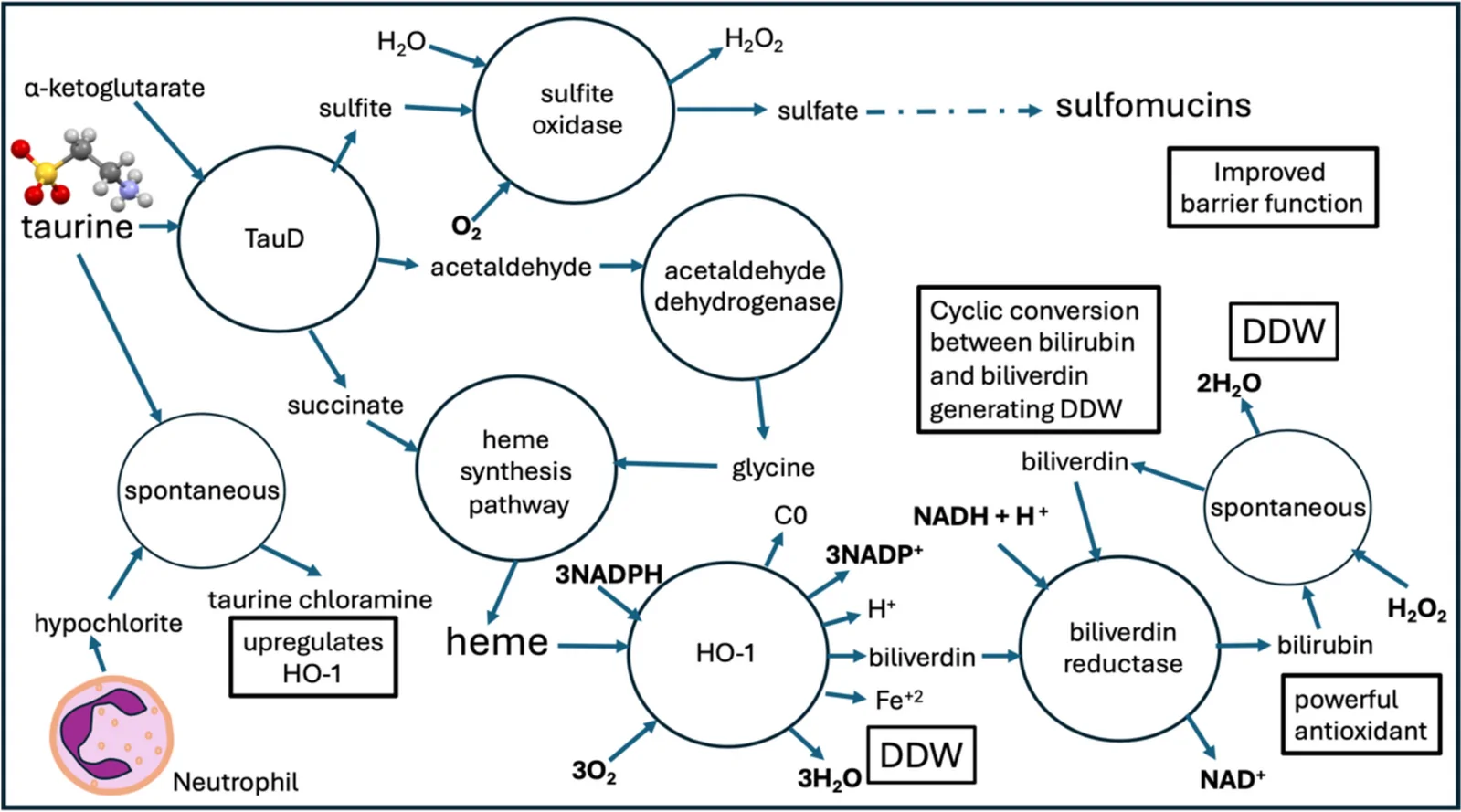
Schematic of possible pathways involving a collaboration between the gut microbiome and the human colonocytes, by which taurine can be processed by specialized microbial enzymes to ultimately yield heme, while N-chlorotaurine (NCT), a spontaneous product of hypochlorite and taurine, can in parallel induce heme oxygenase-1 in response to neutrophil activation following inflammation. This results in the synthesis of bilirubin, a powerful antioxidant, which also produces DDW when it is spontaneously oxidized by H_2_O_2_. *E. coli* can derive sulfate from taurine, which can help support the synthesis of sulfomucins by goblet cells in the colon, enhancing gut barrier function

All these potential reactions are illustrated schematically in Fig. 1. It is intriguing that TauD can produce the precursors to heme, and that NCT activates HO-1, a heme-metabolizing enzyme that helps control damage due to inflammation through the antioxidant properties of its product bilirubin. Both HO-1 and TauD consume oxygen, improving the environment for obligate anaerobes. The sulfate anion that is derived from taurine can help build up sulfomucins, restoring a healthy gut barrier and supporting the production of SCFAs by *A. mucinophila*.

These potential benefits of taurine to gut health could help explain the observation that taurine supplementation protects mice from colon cancer. In a mouse model of colon cancer, mice were treated with azoxymethane (AOM)/sulfate sodium (DSS), which induced colon cancer, and one treated group was also provided with taurine supplementation. Taurine significantly inhibited progression of colon cancer and increased expression of markers of apoptosis as well as the tumor suppressor PTEN (Wang et al. 2020). Similarly, taurine reduces cellular proliferation and induces apoptosis in human colorectal cancer cells (Zhang et al. 2014).

## Recapitulation of the many roles taurine plays in deuterium homeostasis

In this section, we recapitulate key aspects of taurine’s effects in biology that in one way or another support deuterium homeostasis. Taurine supports complex I activity through its conjugation with mitochondrial tRNA. Complex I (NADH dehydrogenase) transfers protons from NADH into the intermembrane space, a crucial step in maintaining an abundant supply of deupleted protons to drive the ATPase pumps. Taurine buffers the alkaline pH of the mitochondrial matrix, which is essential both for β-oxidation of fatty acids and for high catalytic activity of glutathione peroxidase (Hansen et al. 2010). Fatty acids are a low deuterium nutrient. Glutathione peroxidase converts H_2_O_2_ into two molecules of DDW, supporting low deuterium levels in mitochondrial water. The protons in the sulfhydryl groups are deupleted, given that they are derived from NADPH (Rozman 2005).

Taurine detoxifies HOCl produced by immune cells in response to infection. The reaction product, N-chlorotaurine stimulates HO-1, which breaks down heme, producing bilirubin, a potent antioxidant (Olszanecki & Marcinkiewicz 2004; Marcinkiewicz et al. 2009). The reaction catalyzed by HO-1 produces three molecules of DDW, and bilirubin acts, like glutathione, to convert H_2_O_2_ into DDW. Just as for glutathione, bilirubin can produce DDW indefinitely through the chronic recycling between bilirubin and biliverdin (Stocker 2004), capturing a deupleted proton in NADPH to produce a water molecule in each cycle.

Perhaps the most interesting aspect of taurine’s role in deuterium homeostasis has to do with the interaction between gut microbes and the host cells to promote the metabolism of taurine by microbial taurine α-ketoglutarate dioxygenase, ultimately yielding glycine and sulfate as products, along with the conversion of α-ketoglutarate to succinate (Eichhorn et al. 1997). Glycine and succinate are precursors of heme, which can then become useful as a source of deupleted water, as explained above. Sulfate is an essential nutrient for the colonocytes, especially for the synthesis of sulfomucins. Sulfomucins in the glycocalyx support gelled water in the gut barrier, which plausibly exports deupleted protons into the gut lumen. Furthermore, *Akkermansia mucinophila* can metabolize sulfomucins to produce butyrate, a valuable deupleted nutrient to maintain healthy mitochondria in the colonocytes (Earley et al. 2019). Finally, the normal amount of taurine found in cells has been demonstrated experimentally to inhibit DNA damage due to oxidative stress (Messina & Dawson 2000). Thus, taurine protects from oxidative stress caused by mitochondrial deuterium overload.

**Fig. 2:**
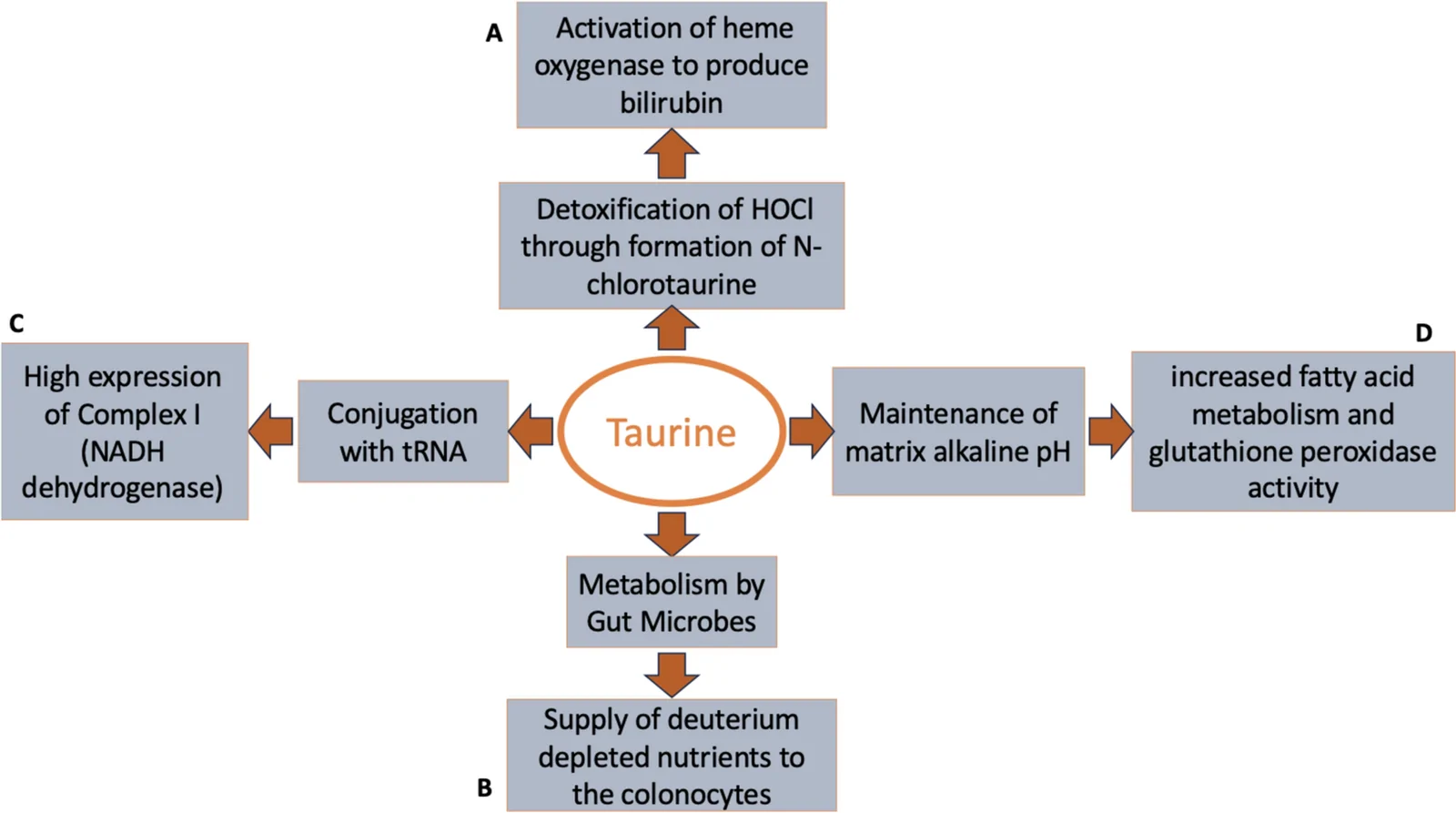
Taurine can help alleviate deuterium toxicity in several ways. (**a**) To support metabolism of heme to yield the antioxidant bilirubin, (**b**) to provide deuterium depleted nutrients through the intestinal microflora, (**c**) to maintain high expression of mitochondrial Complex 1 via its conjugation with tRNA, and (**d**) by maintaining the alkaline mitochondrial matrix pH to support metabolism of deupleted fatty acids and glutathione antioxidant defenses

The key roles of taurine in deuterium homeostasis are illustrated graphically in Fig. 2.

## Conclusions

Taurine is a versatile non-coding amino acid present in high amounts in cells with high energy demand, such as the heart and brain. Taurine is the most abundant amino acid in all human tissues, accounting for 0.1% of total body weight. Comprehensive discussion in previous sections reveals the importance of taurine against ROS and deuterium accumulation in mitochondria.

Human cells readily take up taurine into the mitochondria, where it plays important roles both as a buffer for the alkaline pH of the matrix and, through conjugation with tRNA, to increase synthesis of critical enzymes in respiratory complex I. Taurine conjugation to bile acids may be important for delivery of taurine to the gut microbes. Microbial processing of taurine can potentially yield sulfate to support sulfomucin synthesis and heme, which can be metabolized to the powerful antioxidant bilirubin under conditions of oxidative stress. *β*-oxidation of the SCFA butyrate, a deupleted nutrient, is essential for colonocyte health, and taurine buffering maintains the high pH that optimizes SCFA *β*-oxidation.

Taurine, via its derivative NCT, protects human cells against the toxic bactericidal molecule, HOCl, released by neutrophils. Cancer cells synthesize abundant taurine transporters and rely on taurine to support *β*-oxidation of fatty acids and antioxidant defenses. However, cancer cells are more sensitive to NCT than normal cells. NCT has been shown to cause microbial swelling and apoptosis in lymphoma cells. Multiple studies have shown that taurine supplementation is beneficial in cancer therapy.

A novelty that arises from this investigation is the introduction of the role that deuterium plays in mitochondrial disease, and the ways in which taurine may facilitate the maintenance of low deuterium in the mitochondrial ATPase pumps. Excess deuterium causes a stutter in the pumps, which leads to inefficiencies in ATP production and an increase in ROS. Taurine helps provide deupleted nutrients produced by gut microflora metabolism to maintain deuterium homeostasis throughout the organism. Although taurine can help both normal and cancer cell survival, taurine, especially through its derivative NCT, induces HO-1 which, through excess conversion of NADPH to NADP^+^, may cause insufficient lipid turnover to sustain cancer cell growth.

Taurine, through microbial TauD metabolism, supports production of heme and bilirubin, a powerful antioxidant that protects against colorectal cancer. Bilirubin, produced through taurine/TauD and stimulation of HO-1 via NCT, results in the synthesis of DDW, known for its anti-cancer effects. Taurine can be thought to work in conjunction with DDW treatment as a safe and effective anti-cancer therapy, particularly for colorectal cancer.

## Data Availability

No datasets were generated or analysed during the current study.

## Declarations

### AI assistance

No AI assistance was used for the writing of this paper.
